# Splice it right: the role of PRP21 in regulating seed germination responses to ABA

**DOI:** 10.1093/plphys/kiaf248

**Published:** 2025-06-07

**Authors:** Nilesh D Gawande

**Affiliations:** Assistant Features Editor, Plant Physiology, American Society of Plant Biologists; Department of Biological Sciences and Engineering, Indian Institute of Technology Gandhinagar, Palaj, Gujarat 382355, India

Seed germination is a crucial stage in the life cycle of plants that significantly impacts crop productivity and yield. During this vulnerable stage, plants are highly susceptible to various stress conditions, which can lead to substantial losses in crop production. Various abiotic stress conditions, such as drought, heat, and salinity, affect the seed germination stage, significantly affecting plant life as well as productivity.

**Figure.**
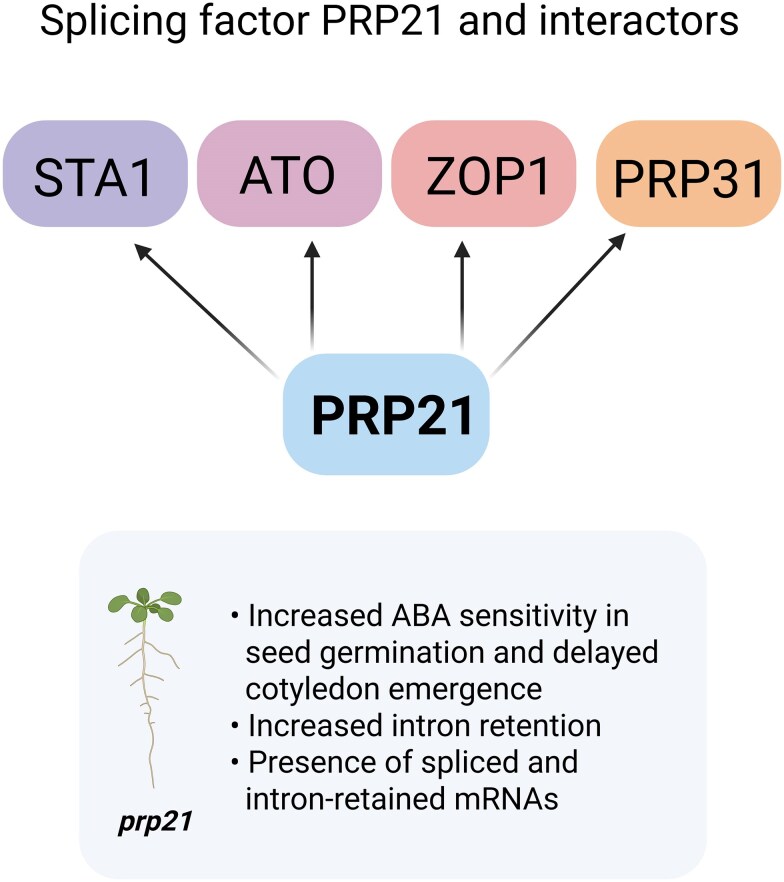
The interacting partners and the effect of splicing factor PRP21 on seed germination in response to ABA in *Arabidopsis thaliana*. PRP21 interacts with other splicing factors (ATO, STA1, ZOP1 and PRP31) and has a critical role in splicing regulation. The *prp21* mutant displayed increased sensitivity to ABA in seed germination. This image was created using BioRender.com.

The plant hormone abscisic acid (ABA) is involved in growth and development, as well as in the reduction of growth in plants. Its effect can vary based on concentration levels, the type of tissue, and the surrounding environmental conditions. ABA has a role in seed germination, and ABA-dependent transcription factors like ABA INSENSITIVE 3 (ABI3) and ABA INSENSITIVE 5 (ABI5) are critical for seed germination and post-germination stages. ABI3 functions upstream of ABI5 and regulates the expression of other ABA-responsive genes (Lopez-Molina et al. 2002). Mutants of these genes are insensitive to ABA, whereas overexpression of these genes increases ABA sensitivity and reduces seed germination.

Alternative splicing is a process that produces multiple protein variants from a single mRNA by combining different segments, which is important in generating protein diversity, particularly in plants facing environmental stresses. The spliceosome is a complex machine that identifies and cuts the mRNA at specific points. Splicing factors are the regulators that either promote or inhibit splicing, which determines gene expression in response to various conditions (Tognacca et al. 2023). In *Arabidopsis*, the splicing factor STABILIZED1 (STA1) and its interacting partners ZINC-FINGER AND OCRE DOMAIN93 ONTAINING PROTEIN 1 (ZOP1) and Pre-mRNA PROCESSING FACTOR 31 94 (PRP31) regulate seed germination as well as stress responses to ABA (Du et al. 2015).

The yeast splicing factor Prp21 and its mammalian counterpart, SF3a120, are important proteins that regulate pre-mRNA splicing. In yeast, Prp21 is an important part of the prespliceosome that interacts with U2 snRNP and pre-mRNA (Arenas and Abelson 1993). Other proteins, such as Prp5, Prp9, and Prp11, work with Prp21 to ensure U2 snRNP binds to pre-mRNA (Ruby et al 1993).

In the present study, Zhou et al. (2025) investigated the role of splicing factor PRP21 in *Arabidopsis thaliana* in seed germination and ABA response. The authors screened the splicing factor mutants involved in ABA responses and found that mutation in the gene *PRP21* leads to ABA sensitivity during seed germination. The *prp21* mutant displayed a significantly lower seed germination percentage compared to wild type in an ABA concentration-dependent manner, and complementation of PRP21 in the mutant restored phenotypes similar to wild type, indicating the critical role of PRP21 in promoting seed germination under ABA. Additionally, *prp21* mutant exhibited a delay in the emergence of green cotyledons compared to the wild type.

The authors further identified interacting protein partners of PRP21 in *Arabidopsis* using affinity purification and mass spectrometry in the *prp21* mutant by expression of FLAG-tagged PRP21. The affinity purification and mass spectrometry results uncovered a number of splicing factors, including ATO (ATROPOS), STA1, and ZOP1. The interactions between PRP21 and PRP31 or ZOP1 were confirmed through co-immunoprecipitation in transgenic plants. Furthermore, the yeast 2-hybrid assay validated the direct interactions between PRP21 with ATO and ZOP1. This highlights the important role of PRP21 in the splicing process through its interaction with other splicing factors.

The *prp21* mutant also exhibited significant changes in intron-retention events, predominantly showing upregulated intron retention compared to wild-type plants, regardless of treatment conditions. Validation through reverse transcription semi-quantitative PCR revealed that wild-type plants had fully spliced mRNAs, whereas the *prp21* mutant displayed both spliced and intron-retained forms, suggesting that restoration of proper splicing occurred with the wild type when PRP21 is present. These findings indicate that PRP21 is a crucial splicing factor for various genes.

The authors further investigated the role of PRP21 in the regulation of gene transcription and mRNA processing in both wild-type and the *prp21* mutant with and without ABA treatment, using Pol II ChIP-seq to assess Pol II presence at genes. They found that *prp21* mutants had increased Pol II presence at many genes, especially at transcription end sites. Poly (A) tag sequencing was also conducted to analyze mRNA polyadenylation. The *prp21* mutant exhibited alternative polyadenylation in other genes compared to wild-type plants, indicating changes in mRNA processing. In conclusion, PRP21 appears to regulate transcription and the processing of the 3′ mRNA end, affecting alternative polyadenylation in response to ABA treatment.

In conclusion, PRP21 is a critical splicing factor in *Arabidopsis thaliana* that promotes seed germination and ABA responses. Future work should investigate whether these splicing mechanisms are conserved across different plant species, which would provide insights into evolutionary adaptations and help plan strategies for enhancing crop resilience to abiotic stresses.
